# Menthol Mouth Rinsing Is More Than Just a Mouth Wash—Swilling of Menthol to Improve Physiological Performance

**DOI:** 10.3389/fnut.2021.691695

**Published:** 2021-07-07

**Authors:** Erica H. Gavel, Kierstyn V. Hawke, David J. Bentley, Heather M. Logan-Sprenger

**Affiliations:** ^1^Faculty of Science, Ontario Tech University, Oshawa, ON, Canada; ^2^Faculty of Health Science, Ontario Tech University, Oshawa, ON, Canada; ^3^Canadian Sport Institute Ontario, Toronto, ON, Canada

**Keywords:** menthol, mouth rinse, hot conditions, exercice, human performance

## Abstract

Interventions that solely act on the central nervous system (CNS) are gaining considerable interest, particularly products consumed through the oral cavity. The oropharyngeal cavity contains a wide array of receptors that respond to sweet, bitter, and cold tastants, all of which have been shown to improve physiological performance. Of late, the ergogenic benefits of carbohydrate (CHO) and caffeine (CAF) mouth rinsings (MRs) have been widely studied; however, less is known about menthol (MEN). That the physiological state and environmental conditions impact the response each product has is increasingly recognized. While the effects of CHO and CAF MRs have been thoroughly studied in both hot and thermoneutral conditions, less is known about MEN as it has only been studied in hot environments. As such, this review summarizes the current knowledge regarding the MEN MR and exercise modality, frequency of the mouth rinse, and mouth rinse duration and compares two different types of study designs: time trials vs. time to exhaustion (TTE).

## Introduction

The topic of fatigue during exercise continues to receive much attention with significant research invested in an attempt to elucidate mechanisms contributing to fatigue and potential solutions to delay fatigue. During exercise, skeletal muscle fatigue may result in a decrease in muscle force generating capacity and performance, especially during endurance exercise in the heat (1, 2). While fatigue is multifactorial, it can be attributed to factors such as reduced metabolic substrates, metabolite accumulation, and cardiovascular mechanisms (3, 4). The role of the brain in regulating fatigue has gained considerable interest in the scientific literature (5), with much debate concerning the regulation of fatigue by the central nervous system (CNS) and the interactions between various physiological and psychological feed-forward and feedback mechanisms (1).

A strategy that has been shown to ameliorate fatigue and improve physiological performance is interventions acting solely on the CNS through an increase in the activity of chemoreceptors and thermoreceptors, specifically, caffeine (CAF), carbohydrate (CHO), and menthol (MEN) mouth rinsings (MRs) (6–8). While all three types of MR have demonstrated physiological benefit (6–8), each has environmental bias. Of late, CHO and CAF MRs have extensively been studied in thermoneutral conditions (7, 8), whereas a MEN MR has been focused on warm or hot conditions (9). Given that changes in environmental conditions, especially high temperatures and great humidity (2, 10), may independently degrade physiological performance, it is important to select an intervention that is environmentally and ecologically valid. Moreover, understanding interventions favorable for certain environments and how they impact physiological performance is pertinent. As such, the purpose of this review is to (1) discuss different types of MR commonly used in sports and (2) explore different variables associated with the MEN MR and improving physiological performance during exercise in the heat.

## Fatigue

Among the variables of fatigue, each characteristic can be categorized as either peripheral or central. Peripheral fatigue is described as a decrease in the muscular force attributed to disruptions with the neuromuscular transmission down the sarcolemma, calcium release and uptake in the sarcoplasmic reticulum, availability of metabolic substrates, accumulation of metabolites, and cross-bridge interactions between actin and myosin (3, 4). Whereas, central fatigue, the inability or failure to continue working at a given exercise intensity, is defined as an activity or exercise-induced decline (progressive reduction) in the activation of a muscle or muscle group (1) and caused by afferent feedback, such as changes in chemoreceptor (11) or thermoreceptor activity, such as exercise in the heat (12).

During exercise, the mechanisms which cause heat-related central fatigue appears to be linked to the CNS (13). While the exact mechanism is unknown, research suggests that central fatigue can be influenced by inhibitory signals sent to the hypothalamus arising from exercise and neurotransmitter networks, affecting brain activity (prefrontal, lateral, orbitofrontal, and anterior cingulate cortexes) (12), specifically the serotonergic and dopaminergic systems (14). The serotonin hypothesis suggests that an exercise-induced increase in extracellular serotonin concentrations contributes to fatigue during exercise (15). Furthermore, the hypothesis also suggests that an increase in the ratio of serotonin to dopamine is associated with feelings of tiredness, whereas a low ratio improves motivation and performance (15). While serotonin has been correlated with feelings of tiredness and lethargy has been suggested to modulate mood, emotion, sleep, appetite, control, and numerous physiological functions (16), dopamine has been suggested to be correlated with feelings of motivation, memory, reward, and attention (15). Many studies have correlated changes in body temperature to a higher perceived effort and a decrease in performance (17). For example, the study by Soares et al. (18), using a rat model, demonstrated that core body temperature was elevated and performance was diminished following an injection of tryptophan, a serotonin precursor. Moreover, research by Lin et al. (19) reported similar results following the administration of fluoxetine (a serotonin reuptake inhibitor) where there was an increase in metabolic heat production and a decrease in heat loss. On the contrary, the study of Watson et al. (20) during exercise in the heat (30°C) demonstrated that dopamine/noradrenaline reuptake inhibitor (bupropion) improved exercise performance following bupropion administration. Taken together, these results suggest that changes in the ratio of serotonin to dopamine can limit and influence physiological performance during exercise in the heat.

## Mouth Rinse Use in Sport

In sports, dietary supplements are often used to improve performance across an array of modalities (21). Depending on the supplement, the absorption time may vary with certain products taking longer time than others (22). For example, when comparing CHO ingestion relative to CAF, it has been suggested that CHO should be ingested at a rate of 1 g/min, or in sufficient amounts, 30 min before fatigue (23), whereas CAF should be consumed 1 h before fatigue as this will allow for plasma CAF concentrations to peak (22). Albeit the positive effects of dietary supplementation during exercise, the ergogenic effects may not be directly proportional to peak oxidation or concentration (24). In light of this, evidence suggests that rinsing of the oral cavity may be used to improve performance through activation of chemoreceptors and thermoreceptors, leading to an increase in brain activity (25, 26). Commonly used MR types in sports include CAF, CHO, and MEN. The purpose of human taste is to enable the appropriate use of chemical cues; these are used in the selection of nutritive, non-nutritive, and toxic foods (27). Taste perception, the sensation produced when a substance reacts chemically with a taste receptor, starts on the tongue and soft palate where the brain processes the stimuli. Moreover, the taste system, known as the gustatory system, acts in concert with the olfactory and trigeminal systems that are responsible for the sense of smell and temperature, respectively (25, 28). The gustatory system, used to differentiate between sweet, salty, sour, and bitter tastants, provides sensory input that is critical for ingestive behavior and toxic compound avoidance; the sense of taste interfaces extensively with neural substrates of reward and motivation (11). Once a gustatory stimulus is evoked, a two-dimensional response discriminative at the cortical level and affective (emotional) at the hypothalamic-limbic level occurs. The discriminative dimension corresponds to the intensity and chemical and physical properties of tastes (29). Among the sensory qualities, each tastant can be denoted as pleasant or unpleasant stimuli; pleasant stimuli will elicit approach and acceptance, while unpleasant ones induce rejection (30). Moreover, each tastant will affect the autonomic nervous system differently. In a study by Rousmans et al. (31), the pleasantly connoted sweet taste induced the weakest electrodermal, thermovascular, and cardiac responses, whereas unpleasantly connoted tastes (salty, sour, and bitter) induced the strongest responses.

Oral temperature is sensed through primary afferent sensory neurons whose cell bodies are located in the dorsal root and the trigeminal ganglia. The signals from these cells are transmitted to the brain *via* the spinal cord, where they are integrated to evoke reflexive and cognitive responses. These sensory neurons are found on the external surface of the body and in the oral cavity and the nose (32). The external surface receptors are involved with thermoregulation, while the receptors in the mouth and nose are involved with the temperature of food and drink (33). The principal molecular thermosensors in the sensory neurons belong to the family of transient receptor potential (TRP)2 channels. So far, six TRP channels have been identified, with four belonging to the TRPV heat-sensing subfamily, TRPV1, TRPV2, TRVP3, and TRVP4, and two belonging to the cold-sensing subfamily, TRMP8 and TRPA1. Some of these channels are also sensitive to compounds that mimic temperatures (34, 35). The TRPV1 channels in sensory nerves respond to heat and capsaicin, an alkaloid from “hot” peppers, which binds to open the channel and thus depolarizes the neuron and fires action potentials (36). The brain interprets this information as an increase in ambient temperature and initiates vasodilation and sweating. TRPM8, which binds ligands like MEN or icilin and elicits a cold sensation, is a non-selective cation channel predominantly expressed in a subpopulation of thermoceptive/nociceptive neurons found in the dorsal root and the trigeminal ganglia. From the trigeminal ganglion, increased activity in the insular taste cortex, the somatosensory cortex, the orbitofrontal cortex, the anterior cingulate cortex, the ventral striatum, and the pregenual cortex is observed (25). Stimulation of this thermoreceptor can lead to shivering, a mechanism to raise body temperature. TRPM8 is activated by cold temperature with a threshold of ~22°C (37, 38) and by MEN (39).

During exercise, it has been suggested that oral receptors within the mouth directly stimulate reward centers in the brain, which increase “central drive” and improve work capacity: this has been observed in CHO, CAF, and MEN MRs (11, 24, 40–44). The activation of reward areas in the brain, such as the insula/frontal operculum, orbitofrontal cortex, and striatum, is suggested to lower the perception of exertion during exercise (11, 45) and, potentially, feelings of displeasure (46). Through the receptors in the mouth, it is speculated that CHO, CAF, and MEN enter the brain *via* different pathways (25, 47). While MEN activates the oropharyngeal TRPM8 thermoreceptors, CHO and CAF interact with the gustatory chemoreceptors, T1R2/ T1R3 sweet and TAS2R bitter receptors, respectively. It was originally proposed that a CAF MR elicited its effects by allowing CAF molecules to inhibit adenosine through binding of the adenosine receptors (48, 49); however, Doering et al. (50) reported that a CAF MR does not increase blood CAF concentrations. Moreover, the same response is observed with the CHO MR (51). An electroencephalography recording which has shown that while a CAF MR increases activity among the orbitofrontal and dorsolateral prefrontal cortex, a CHO MR only increases activity in the orbitofrontal cortex (47) suggests that there may be a summation effect of CAF and CHO. This postulation was debunked in a high-intensity running study whereby the summation effect of CHO + CAF MR did not significantly improve performance compared to CAF alone (52).

### Effects of MEN MR

Menthol presents in nature both as a fragrance and as a flavor molecule targeting the olfactory and gustatory systems which impart feelings of coolness and freshness (53). MEN has historically been found in an array of products, such as candies, chewing gums, toothpastes, common cold medications, vaporubs, cigarettes, and aromatherapy medications, but a more contemporary application of MEN has been that of an ergogenic aid that can be applied topically, used as a mouth swill, or ingested alongside ice slurry (54). Perhaps, most promising is that, when used as a mouth rinse, MEN is shown to increase the drive to breathe, elevate ventilation, increase arousal, and attenuate thirst, as well as elicit sensations of coolness and freshness that may alleviate thermal symptoms during exercise (32, 55). Past research indicates that MEN has the capability to increase self-selected cycling power output (56) and increase cycling TTE when rinsed in the mouth (57) *via* stimulation of oropharyngeal cold receptors. This suggests that afferent signals emanating in the oral cavity are capable behavioral controllers (9). More specifically, when swilled, MEN, activates TRPM8 (34, 35). As previously stated, TRPM8 is the primary molecular transducer of cold somatosensation and therefore is shown to be associated with improved thermal comfort, reduced ratings of perceived exertion (RPE), and improved performance during exercise in hot conditions (9, 57, 58). Since the excitability of the cerebral cortex is controlled by the brainstem reticular formation (59), MEN is understood to be a stimulus that can influence the level of consciousness/arousal of an individual *via* stimulation of the trigeminal nerves (32). The trigeminal nerve is a cranial nerve, composed of three major branches of nerves, namely, the ophthalmic, maxillary, and mandibular nerves, that converge on the trigeminal ganglion, located within Meckel's cave. The trigeminal ganglion also contains the cell bodies of incoming sensory nerve fibers that make them responsible for providing sensations to the face, mucous membranes, and other structures of the head (32, 60). Additionally, it is known that motor fibers pass through the trigeminal ganglion without synapsing on their way to peripheral muscles. This implies that MEN is effective at reducing thermal sensation and/or state *via* its action on the trigeminal ganglion, which may contribute to increased skeletal muscle activation during hyperthermic exercise (60). Moreover, it has been suggested that, when MEN stimulates the trigeminal system, this directly activates reward/pleasure centers in the brain to increase “central drive” and improve work capacity (11, 24, 40, 42–44). The activation of reward areas in the brain, such as the insula/frontal operculum, the orbitofrontal cortex, and the striatum, is suggested to lower the perception of exertion during exercise (11, 45) and, potentially, feelings of displeasure (46). A study conducted by Guest et al. (25) introduced various temperatures of artificial saliva into the mouth and recorded activation of various brain regions and perceived pleasantness. Investigators found that a cold fluid (5°C) was perceived to be more pleasant when compared to a warm (50°C) solution and that some of the brain regions involved in detecting temperature were involved in sensing pleasantness. This research shows that pleasant stimuli can help to maintain central drive and increase motivation during exercise performance (25). However, evidence suggests that the magnitude of performance increment is dependent on several factors and must be taken with caution. Finally, in line with the central fatigue hypothesis (15), a study by Guest et al. (25) also showed that intra-oral thermal stimulation activates the network of taste- and reward-responsiveness regions of the human brain that is associated with dopaminergic pathways within the primary and secondary cortices. Since serotonergic and dopaminergic systems pervasively interact with each other, modulating the serotonin-to-dopamine ratio appears to be significant for determining fatigue and regulating physical performance outcomes (25, 61). Given the well-defined role of dopamine in the initiation of movement, it is likely that adaptations in dopaminergic pathways influence exercise capacity (62). These are the same regions engaged with positive self-talk and which indicate that MEN could be linked to an increase in dopaminergic activity among the reward centers of the brain (61). This implies that an increase in the neurotransmission of the dopaminergic pathway improves the activation of the basal ganglia and increases stimulation of the motor cortex, which then reduces the effects of central fatigue and benefits aerobic performance (58, 62).

### Performance Outcomes With MEN

According to the published literature, the use of MEN has demonstrated performance improvements in 8 of 10 published studies (9, 54, 57, 58, 63–68) at different drink temperatures and frequencies (55, 67) in time trials (TTs) and endurance events (57, 66), at concentrations of 0.01% and 0.05% (57, 65), and in females (68). Thus, when applied internally as an ergogenic intervention, MEN appears to improve overall exercise performance in the heat, as well as effectively lowering TS (*p* < 0.001) (6), *via* its anticipated ability to ameliorate the effects of impaired muscle activation caused by central fatigue (2) or anticipatory afferent signals to the brainstem (69). This section of the review provides an analysis of performance change outcomes (%) across various investigations involving MEN. While MEN has demonstrated physiological improvements under hot conditions and in a male population, the influence of MEN in both thermoneutral and cold conditions is unknown. Moreover, the effect of MEN, or in concert with other products, in the female population has not been explored.

### Exercise Modality and Use of Menthol

Of late, the influence of MEN has been investigated in cycling, running, isometric contractions, and vertical jumps (58, 63, 66). While the influence of MEN appears to have no performance benefit in isometric contractions and vertical jumps (63), MEN has shown benefits in both running and cycling studies (70). Given that a MEN MR acts solely on the CNS, this is of no surprise as decrements in performance for short-duration exercise are more likely linked to peripheral vs. central fatigue (71).

In contrast, while no study has compared the effects of a MEN MR in running and cycling, based on a meta-analysis by Jeffries and Waldron (6), it appears that the improvement of performance is slightly higher in cycling relative to running, with values of 6.7 ± 1.4 and 4.4 ± 1.6%, respectively; however, the MEN MR protocol was significantly different between studies. Although exercise modality may impact how one might respond to the MEN MR, it should be noted that all of the running studies done with the MEN MR consisted of a TT vs. a combination of TT and time to exhaustion (TTE) work observed in the cycling studies. Moreover, the frequency of the MEN MR also differed between modalities. For example, in the studies based on running by Stevens et al. (66) and Stevens et al. (54), the MEN MR was administered once every 200 m, whereas studies based on cycling by Mundel and Jones (57), Flood et al. (58), and Gibson et al. (64) administered the MEN MR once every ~10 min. With regard to participants, all studies used recreationally active males, so the outcomes should not be attributed to the skill level of the participants. As such, further research warrants investigation to compare modalities of similar exercise durations and MEN swilling protocols.

### Time to Exhaustion vs. Time Trial

It is well-established that a MEN MR can improve physiological endurance performance during exercise in hot conditions; however, it appears that the test protocol may play a significant role in the rate of improvement following a MEN MR (9, 54, 57, 58, 64, 66–68). For example, Mundel and Jones (57) reported an improvement of 9% in a cycling TTE, whereas Tran Trong et al. (67) only saw an improvement of 6.2% in a time trial. Furthermore, in other TTE studies by Flood et al. (58) and Jeffries et al. (9), they saw improvements of 7 and 6%, respectively, while TT improvements were again lower in studies by Riera et al. (65) (5.3%), Stevens et al. (66) (3%), Stevens et al. (54) (4%), and Gibson et al. (64) (2.3%). Although intra-subject variance is much higher in a TTE vs. a TT study, >10 and <5%, respectively (72), each study, except that by Tran Trong et al. (67), included a familiarization trial to help reduce the amount of variation between trials. Furthermore, given that the total amount of exercising time did not significantly differ among modalities, with 35.3 ± 19.7 min for the TTE (9, 57, 58), and 42.6 ± 25.8 for the TT, the duration of the trial and time in the heat should have influenced the impact of MEN on performance.

### Frequency of MEN MR

While the frequency of a MEN MR has not been thoroughly explored, the most common protocol includes one MR per minute studied entirely on running (54, 66), and once every ~10 min (57, 65, 68, 73, 74). Furthermore, a study by Jeffries et al. (9) had the participants do one MR during the latter stages of exercises (at 85% of TTE), whereas Tran Trong et al. (67) had a group of trained runners consume 190 ml of a MEN beverage throughout exercise, during warm-up, every block, and recovery. Although the frequency was different among the present studies, MEN improved performance in each study with a rate of ~3.5% in one MR per minute (54, 66), ~4.5% once every 10 min (57, 58, 64, 65), and ~6% when consumed at 85% of TTE (9, 67). Of note is the test protocol, during which MEN concentration, swilling duration, and exercise modality were different among each study. Future research should explore the influence of MEN MR frequency in the latter stages of exercise when central fatigue is traditionally high (75).

### MEN Swilling Duration and Consumption

Of late, the most common use of oral MEN is intermittent consumption throughout (65, 67) and swilling. Although swilling duration has not been studied, popular durations include 10 (57) and 5 s (9, 54, 58, 64, 66, 68). While consumption has demonstrated the greatest performance benefits (improvement of ~5.8%), both 5 and 10 s of repeated MEN MR has displayed improvements of 9.5 and 3.7% in cycling and running performance, respectively. Despite only one study using a 10 s MR duration (57), Sinclair et al. (76) suggest that a 10 s CHO MR is more beneficial than a 5 s. Although both swilling durations improved endurance performance relative to a placebo, the 5 s MR was not significant, whereas the 10 s MR was significant (*p* ≤ 0.01) (76). Furthermore, a study by Stevens et al. (66) comparing ice-slurry ingestion relative to the MEN MR suggests that swilling duration does have a significant impact as the MEN MR improved performance whereas the ice-slurry ingestion did not. Moreover, the studies by Sinclair et al. (76) provide evidence that swilling duration (the exposure time of the MEN in the oral cavity) may influence performance, with longer swilling times being more beneficial.

### Thermal Sensation

Despite multiple studies indicating a negligible improvement in TS with MEN MR, there are a few studies demonstrating a negative relationship between MEN MR and thermal perception while proposing that the change in thermal sensation with MEN MR accounts for one of the mechanisms of MEN use for the improvement of endurance performance (6). To illustrate, studies by Flood et al. (58) and Jeffries et al. (9) saw a significant decrease in thermal sensation for a given workload with the MEN MR; however, research by Riera et al. (65), Stevens et al. (66), and Gavel et al. (68) observed no difference. While all studies had trials completed under hot conditions, research by Flouris and Cheung (77) suggests that the differences among each study could be due to day-to-day variability vs. the actual intervention. Using a modified model by Gagge et al. (78), it was reported that differences in thermal sensation and thermal comfort were not observed in tandem with an increase in core body temperature and skin temperature. Moreover, in a systematic review by Koelblen et al. (79) comparing seven thermal sensation models, there were differences between each model for the same level of environmental exposure. Based on this, one may conclude that a lack of validity among thermal scaling models during exercise could influence the results with regard to the effect of a MEN MR on thermal perception.

### Ratings of Perceived Exertion

Of late, the influence of MEN on the Rating of Perceived Exertion (RPE) is ambiguous and needs further investigation. To illustrate, Mundel and Jones (57), Flood et al. (58), Jeffries et al. (9), and Gavel et al. (68) reported that the RPE changed for a given workload with the MEN MR, while research by Riera et al. (65), Tran Trong et al. (67), Stevens et al. (66), and Stevens et al. (54) did not. Of note, all studies shared similar environmental conditions (~34°C and RH 40%), which is an important factor as research has demonstrated the influence of the environment on how one modulates motor output (80). Factors not consistent between each study were frequency, concentration, and duration at which the MEN MR was administered. Although the frequency and duration of MEN MR have never been tested, one may speculate that this could impact performance (53). Relative to the CHO MR, the MEN MR demonstrates an acute increase in the activity of the reward centers of the brain, the proposed mechanism for the improvement of performance with MEN (25, 26, 32). In support, Guest et al. (25) demonstrated acute differences in the reward centers of the brain when comparing warm and cold water. Similarly, Smeets et al. (26) showed similar results when comparing the difference in CHO concentrations. Thus, the impact of manipulating the MR protocol (frequency, concentration of MEN, duration, etc.) may have significant effects on the efficacy of MEN as a non-thermic aid while suggesting that the differences between each study may be due to the variability in the MR protocol. Moreover, there are many questions left unanswered when examining the current MEN MR literature and the link of MEN to performance improvements.

## Conclusion

Menthol MR appears to improve performance in moderate- and high-intensity exercises of ~20 to >60 min. It is suggested that the mechanism associated with the improvement of performance is related to the CNS *via* oral cold receptors that activate the reward centers of the brain. While the effects of MEN MR have not been tested in thermoneutral conditions, these oral cold receptors seem to be responsive in hot conditions, at high core temperatures, and during cycling and running exercises. Given that MEN is easily transportable, low in cost, and accessible at most grocery and convenience stores, MEN MR might be a viable non-thermic alternative to the external application of cooling devices used to improve performance during exercise in the heat.

## Author Contributions

All authors provided substantial contributions to the conception and design of the work, drafting the work, and revising it critically. All authors provided final approval for the submitted/published version and provided consent for publication.

## Conflict of Interest

The authors declare that the research was conducted in the absence of any commercial or financial relationships that could be construed as a potential conflict of interest.
